# Sex differences in [^11^C]ABP688 binding: a positron emission tomography study of mGlu5 receptors

**DOI:** 10.1007/s00259-018-4252-4

**Published:** 2019-01-09

**Authors:** Kelly Smart, Sylvia M. L. Cox, Stephanie G. Scala, Maria Tippler, Natalia Jaworska, Michel Boivin, Jean R. Séguin, Chawki Benkelfat, Marco Leyton

**Affiliations:** 1 Department of Psychiatry, McGill University, 1033 Pine Avenue West, Montreal, QC H3A 1A1 Canada; 2 Department of Cellular and Molecular Medicine, University of Ottawa, Ottawa, ON K1H 8M5 Canada; 3 Institute of Mental Health Research, affiliated with the University of Ottawa, Ottawa, ON K1Z 7K4 Canada; 4 Department of Psychology, Université Laval, Quebec City, QC G1V 0A6 Canada; 5 CHU Ste-Justine Research Center, Montreal, QC H3T 1C5 Canada; 6 Department of Psychiatry and Addiction, Université de Montréal, Montreal, QC H3T 1J4 Canada; 7 Department of Neurology & Neurosurgery, Montreal Neurological Institute, McGill University, Montreal, QC H3A 2B4 Canada; 8 Department of Psychology, McGill University, Montreal, QC H3G 1G1 Canada; 9 Center for Studies in Behavioral Neurobiology, Concordia University, Montreal, QC H4B 1R6 Canada

**Keywords:** Metabotropic glutamate receptors, mGluR5, PET, Gender

## Abstract

**Purpose:**

The purpose of this study was to assess, in a large sample of healthy young adults, sex differences in the binding potential of [^11^C]ABP688, a positron emission tomography (PET) tracer selective for the metabotropic glutamate type 5 (mGlu5) receptor.

**Methods:**

High resolution [^11^C]ABP688 PET scans were acquired in 74 healthy volunteers (25 male, 49 female, mean age 20 ± 3.0). Mean binding potential (BP_ND_ = *f*_ND_ * (B_avail_ / K_D_)) values were calculated in the prefrontal cortex, striatum, and limbic regions using the simplified reference tissue model with cerebellar grey matter as the reference region.

**Results:**

[^11^C]ABP688 BP_ND_ was significantly higher in men compared to women in the prefrontal cortex (*p* < 0.01), striatum (*p* < 0.001), and hippocampus (*p* < 0.05). Whole-brain BP_ND_ was 17% higher in men. BP_ND_ was not related to menstrual phase in women.

**Conclusions:**

Binding availability of mGlu5 receptors as measured by PET [^11^C]ABP688 is higher in healthy men than women. This likely represents a source of variability in [^11^C]ABP688 studies and could have relevance for sex differences in cognitive-behavioral functions and neuropsychiatric disorders.

## Introduction

ABP688 (3-((6-methylpyridin-2-yl)ethynyl)cyclo-hex-2-en-1-one-O-methyloxime) is a selective allosteric ligand of the metabotropic glutamate type 5 (mGlu5) receptor. Positron emission tomography (PET) studies with ^11^C labeled ABP688 have identified replicable group differences [1–4], but variability in the tracer’s binding in humans has proven to be unexpectedly high [5, 6]. Some sources of variability have been identified, including circadian changes in receptor availability [6, 7] and tracer *(E)*-isomer content [8]. However, variability remains high when these factors are accounted for [5, 8], suggesting that further sources remain unknown.

One potential source of variability is biological sex. Lower PET [^11^C]ABP688 binding in healthy women compared to men was seen in one study [1], but this was not found in a more recent comparison [9]. In clinical populations, the majority of scans have been conducted in men, but some evidence has emerged of sex-specific disease effects. In people with schizophrenia, regional [^11^C]ABP688 BP_ND_ was higher in female patients but lower in male patients relative to sex-matched healthy controls [10]. Pre-clinical and post-mortem research also suggests that sex differences exist in the role of mGlu5 receptors in substance use disorders, depression, and responses to stress [11–14].

Given the high variability in tracer binding measures, the relatively small sample sizes in previous studies coupled with the possibility of menstrual-cycle associated changes might have limited the ability to detect sex differences. Thus, the current study’s objective was to assess sex differences in [^11^C]ABP688 BP_ND_ in a large sample of healthy young adults.

## Methods

Seventy-four healthy volunteers were included in this study (25 men and 49 women, mean age 20 ± 3.0 years). Five participants were current cigarette smokers (4 women, 1 man); none of the participants had any Axis I psychiatric disorders. Participants were recruited from community advertisements (*n* = 25) or from one of three longitudinal cohorts (Quebec Study of Newborn Twins, *n* = 5, and two cohorts from the Quebec Longitudinal Study of Child Development, *n* = 44). In the case of twins, only a single volunteer per twin pair was included. The study was carried out in accordance with the Declaration of Helsinki and approved by the Research Ethics Board of the Montreal Neurological Institute, McGill University, the ethics committee of the CHU Sainte-Justine Research Center, and the ethics committe of the Institut de la Statistique du Quebec. All participants provided written informed consent.

For female participants, menstrual phase at the time of the scan was determined based on the date of last menstrual period and length of cycle (self-report). Serum levels of luteinizing hormone (LH) and follicle-stimulating hormone (FSH) were measured in a subset of participants (*n* = 10) to confirm this. Of women not using hormonal contraception (*n* = 29), the majority (*n* = 21) were tested during the follicular phase, five during the luteal phase, and three during ovulation.

PET scans were acquired between 10 am and 3 pm using a high-resolution research tomograph (HRRT, CTI/Siemens). Prior to injection of the ligand, a 6-min transmission scan was performed with ^137^Cs to correct for tissue attenuation. Subsequently, a 60-min dynamic scan was initiated concurrent with the beginning of a one-minute bolus injection of 370 MBq [^11^C]ABP688. Dynamic data were collected with the scanner in list mode and reconstructed using an ordered subset maximization algorithm including motion correction to the transmission scan. High-resolution (1 mm^3^) T1-weighted anatomical magnetic resonance imaging (MRI) scans were acquired using a 1.5 T Siemens Sonata scanner (gradient echo pulse sequence, repetition time = 9.7 ms, echo time = 4 ms, flip angle = 12°, field of view = 250 mm and matrix = 256 × 256) or a 3 T Siemens Trio TIM scanner (MPRAGE sequence, repetition time = 2300 ms, echo time = 3.42 ms, flip angle = 9°, field of view = 256 mm and matrix = 256 × 256).

Regions of interest (ROIs) were defined using standard masks on the MNI152 template then registered to individual PET images. The ROIs included three prefrontal cortex subregions (orbitofrontal, dorsolateral, and medial), three functional striatum subregions (associative, sensorimotor and ventral), insula, hippocampus, and amygdala. Regional non-displaceable binding potential values (BP_ND_) were extracted from each ROI using the simplified reference tissue model with cerebellar grey matter as the reference tissue. Scan start times were compared between men and women using independent samples *t* tests. Percent *(E)*-isomer content and injected tracer mass were compared using the Wilcoxon rank sum test due to their non-normal distribution. The effect of sex on BP_ND_ values was analyzed using repeated measures analysis of covariance (ANCOVA) with region as a repeated measure, sex as a between subject factor, and tracer *(Z)*-isomer content and smoking status as covariates. Post-hoc independent samples *t*-tests were then performed within each ROI. Whole brain voxel-wise analyses of BP_ND_ were compared using SPM12 (Wellcome Functional Imaging Laboratory). Summary BP_ND_ values were computed as the unweighted mean of all examined regions. One-way ANOVA was used to assess the effect of menstrual phase on BP_ND_. In exploratory analyses, correlations between summary BP_ND_ and serum LH or FSH levels were assessed using Pearson’s *r*.

## Results

Sample and PET scan characteristics are summarized in Table 1. Scans performed on men and women did not differ in start time (*t* = −0.001, *p* = 1.0) or % *(E)*-isomer in tracer batch (Wilcoxon rank sum test W = 708, *p* = 0.28). Tracer injected mass was higher in scans performed on women than men (W = 437, *p* = 0.045). Injected mass was not correlated with BP_ND_ in any region (ps > 0.06, uncorrected) and was therefore not included in subsequent analyses [15].

**Table 1:** Participant and scan characteristics

Characteristic	Men	Women	*p*-value
Age (mean ± SD)	20.7 ± 4.2	19.6 ± 2.2	*0.13*
Smokers (n)	1	4	*0.66*
Recruitment method (n)	9 CA, 16 QCS	16 CA, 33 QCS	*0.98*
Scan start time, minutes from 10:00 (mean ± SD)	102 ± 56.2	102 ± 58.1	*1.0*
% *(E)*-isomer (mean ± SD)	91.6 ± 4.9	91.8 ± 3.2	*0.23*
Mass tracer injected, μg (mean ± SD)	7.07 ± 6.1	10.2 ± 6.6	*0.045*

CA, community advertisement; QCS, Quebec cohort studies (Quebec Longitudinal Study of Child Development, *n* = 44 or the Quebec Study of Newborn Twins, *n* = 5)

*p* values from Fisher’s exact test (smokers), Chi-squared test (recruitment method), independent samples *t*-tests (age and start time), or Wilcoxon rank sum tests in the case of non-normality (isomer content and tracer mass)

**Fig. 1:**
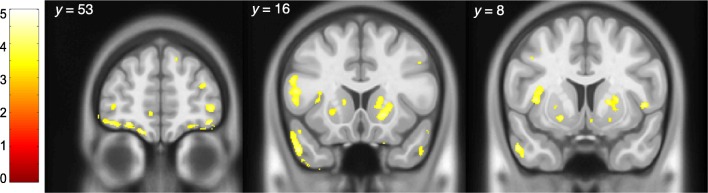
Voxel-wise *t*-map showing higher [^11^C]ABP688 BP_ND_ in men compared to women (threshold *t* = 3.21)

In the ROI analysis of BP_ND_, the ANCOVA yielded main effects of sex (F_1,68_ = 12.8, *p* = 0.001) and region (F_8,544_ = 3.6, *p* < 0.001) and a sex × region interaction (F_8,624_ = 6.0, p < 0.001). In post hoc comparisons of each ROI, BP_ND_ was significantly higher in men compared to women in all regions (*p*s < 0.042) apart from the amygdala (*p* = 0.062) (Table 2). Comparison of the magnitude of sex differences in each region suggests that the sex × region interaction emerges from greater difference between sexes in prefrontal cortex subregions than in subcortical limbic structures. The largest magnitude of difference was in the orbitofrontal cortex where mean BP_ND_ was 22% greater in men compared to women, and the smallest in the amygdala, where BP_ND_ was 11% higher in men. Voxel-wise analyses were consistent with these findings, with clusters of higher BP_ND_ in men emerging in the prefrontal cortex, striatum, and insula (Fig. 1), as well as in the temporal, parietal, and occipital cortices (cluster-level *p*s < 0.05, familywise-error-corrected).

**Table 2:** Mean [^11^C]ABP688 BP_ND_ is higher in men than in women across the brain

Region	Men, mean ± SD BP_ND_	Women, mean ± SD BP_ND_	% Difference (men > women)	*p*-value
mPFC	1.1 ± 0.26	0.94 ± 0.17	16%	0.0097
dlPFC	1.0 ± 0.25	0.83 ± 0.16	20%	0.0045
OFC	0.95 ± 0.22	0.78 ± 0.14	22%	0.00093
Associative striatum	1.3 ± 0.22	1.1 ± 0.18	16%	0.00068
Sensorimotor striatum	1.0 ± 0.18	0.86 ± 0.14	17%	0.00016
Ventral striatum	1.4 ± 0.24	1.2 ± 0.18	17%	0.00029
Insula	1.3 ± 0.21	1.1 ± 0.17	17%	0.000081
Hippocampus	0.72 ± 0.18	0.64 ± 0.14	13%	0.041
Amygdala	0.76 ± 0.18	0.69 ± 0.15	11%	0.062
**Summary BP**_**ND**_	**1.0 ± 0.20**	**0.89 ± 0.15**	**17%**	**0.00037**

mPFC, medial prefrontal cortex; dlPFC, dorsolateral prefrontal cortex; OFC, orbitofrontal cortex; *p* values from two-tailed t-tests. Summary BP_ND_ was computed as the unweighted mean of all regions

**Fig. 2:**
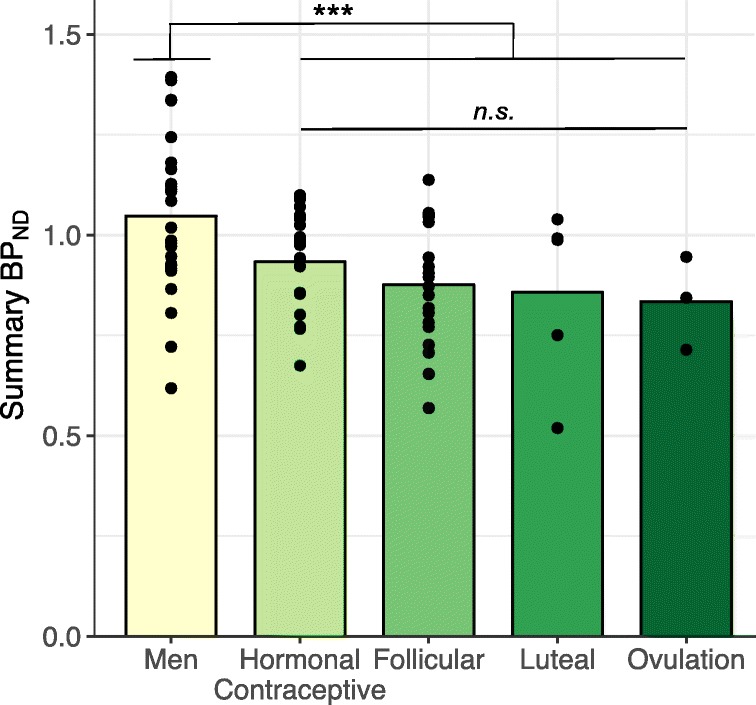
Mean BP_ND_ values across regions are higher in men compared to women but did not differ across menstrual phase in women (F_1,47_ = 2.4, *p* = 0.13); *** indicates *p* < 0.005

In women, BP_ND_ values did not differ across menstrual phase groups (*p*s > 0.05 in each region) (Fig. 2). BP_ND_ was not statistically related to LH (*r* = −0.33, *p* = 0.35) or FSH (*r* = −0.043, *p* = 0.91) levels in a subset of ten women.

## Discussion

In this pooled analysis of healthy young adults comprising the largest sample reported to date, brain [^11^C]ABP688 BP_ND_ values were significantly higher in men compared to women.

These results are in agreement with a previous study finding lower [^11^C]ABP688 BP_ND_ in female nonsmokers compared to male nonsmokers [1]. In comparison, several studies with an even sex split or greater numbers of female participants found no effect of sex on tracer binding [2, 9, 16]. Given the high variability in [^11^C]ABP688 binding estimates, sex differences may have been masked in previous studies with smaller sample sizes.

The observed sex differences might reflect an influence of gonadal hormones. Estrogen receptors are functionally coupled to mGlu5 receptors in striatal medium spiny neurons, and treatment with a negative allosteric modulator for mGlu5 abolishes estradiol enhancement of stimulant sensitization [12, 17]. The present work suggests that sex differences are present in healthy humans and can be identified using PET [^11^C]ABP688 imaging. Future studies should account for these differences both as a possible source of variability in binding measures and as a biological factor potentially contributing to sex differences in neurocognitive function and neuropsychiatric disorders.
